# Is There a Role for Large Exome Sequencing in the Management of Metastatic Non-Small Cell Lung Cancer: A Brief Report of Real Life

**DOI:** 10.3389/fonc.2022.863057

**Published:** 2022-03-07

**Authors:** Lorraine Dalens, Julie Niogret, Courèche Guillaume Kaderbhai, Romain Boidot

**Affiliations:** ^1^Medical Oncology Department, Georges-François Leclerc Cancer Center – UNICANCER, Dijon, France; ^2^Molecular Biology Unit, Department of Biology and Pathology of Tumors, Georges-François Leclerc Cancer Center – UNICANCER, Dijon, France; ^3^UMR CNRS 6302, Dijon, France

**Keywords:** lung adenocarcinoma, small NGS panel, large NGS panel, exome analysis, utility

## Abstract

**Introduction:**

Non-small-cell lung cancer (NSCLC) is one of the main causes of death by cancer worldwide. With the rise of targeted therapies, the search for molecular abnormalities is becoming a crucial step in the management of lung cancer. Whole exome sequencing (WES) is developing rapidly and is now accessible in routine care. However, its value, compared to smaller gene panels, remains unclear.

**Methods:**

We conducted a retrospective analysis of all 281 patients with lung carcinoma referred to the Molecular Tumor Board of the Georges-François Leclerc Cancer Center (CGFL) between March 2015 and January 2018. We compared the results of standard molecular testing with the results of WES performed on every patient.

**Results:**

WES highlighted many more mutations than smaller panels (mutations were found in 82 genes, while smaller panels found, at the most, mutations in 12 genes). Most of these mutations were class III or IV according to the ESCAT classification. The exome sensitivity also showed limitations, notably a slightly lower efficiency for common mutations, including classical EGFR mutations.

**Conclusion:**

Small, targeted panels could be preferred over WES at the initial diagnosis of metastatic NSCLC. They are more sensitive for the identification of mutations on the most frequently mutated genes, such as *ALK, BRAF, EGFR, ERBB2, KRAS* or *MET*. Larger panels or WES could be useful at disease progression, to enlarge treatment possibilities by highlighting uncommon but potentially targetable mutations that are not covered by smaller, targeted panels.

## Introduction

With over 2 million cases per year, lung cancer is the second most frequent cancer worldwide. It is the leading cause of death by cancer (18.4% of all cancer deaths).

The first-line treatment in metastatic NSCLC is based on chemotherapy associated with immunotherapy or immunotherapy alone, depending on the level of PD-L1 expression. However, in the presence of an oncogenic driver, targeted therapies are surpassing this strategy. An activating *EGFR* mutation or *ALK/ROS1* rearrangement is present in around 15% of Caucasian patients with NSCLC. Many other drivers are potentially targetable, making the search for molecular abnormalities a key feature during the diagnosis.

Almost every NSCLC with an oncogenic driver is an adenocarcinoma. Therefore, international guidelines recommend molecular testing for all patients with advanced adenocarcinoma. Testing *EGFR* mutations and *ALK* and *ROS1* rearrangements has become mandatory. In addition, other promising molecular alterations, such as *BRAF, MET, ERBB2, RET* and even *KRAS*, should be investigated at diagnosis because of the rapid therapeutic advances.

Multiple panels of genes are available to determine the patient’s molecular status.

In recent years, next-generation sequencing (NGS) has revolutionized cancer treatment. NGS can be used to screen small targeted gene panels, or very large panels, including whole exome sequencing (WES).

WES has demonstrated its feasibility in routine cancer care (1). As suggested by the MOSCATO trial, patient outcome could be improved by increasing the possibility to find targetable mutations (2).

In 2018, the ESMO Precision Medicine Working Group recommended using NGS for patients with advanced non-squamous cell carcinoma, in order to detect ESCAT (ESMO Scale For Clinical Actionability of molecular Targets) level I alterations. They do not recommend larger panels detecting alterations with a lower level of evidence in first-line (3).

Therefore, the role for WES in the treatment strategy of NSCLC remains unclear.

In this study, we present a retrospective cohort of 281 patients with metastatic NSCLC, who had molecular testing at diagnosis, and large exome sequencing analyzed by a Molecular Tumor Board (MTB). The primary objective was to determine the role of large exome sequencing during the treatment of metastatic NSCLC.

## Materials and Methods

All 281 patients with lung carcinoma referred to the Molecular Tumor Board of the Georges-François Leclerc Cancer (CGFL) Center between March 2015 and January 2018 were included in this retrospective analysis. They were all included in the EXOMA (NCT02840604) or ALCAPONE (NCT02281214) trials. This study on patient samples was conducted in accordance with the Declaration of Helsinki and approved by the Ethics Committee of the Georges-François Leclerc Cancer Center (Dijon, France) under the number 00010311, and by the Consultative Committee of Burgundy (Dijon, France) for the Protection of Persons Participating in Biomedical Research (Comité Consultatif de Protection des Personnes en Recherche Biomédicale de Bourgogne). Written informed consent was provided.

For all 281 patients, we compared the results of different panels: large exome sequencing, an in-house panel developed by the CGFL and a commercial panel (the latter two are hereafter referred to as “small panels”), and hotspot analysis.

Every patient had molecular testing at diagnosis (hotspot or small panel) and WES. If a panel was not performed for a particular patient, but another panel or WES found a mutation in a gene covered by the non-performed panel, then we considered that the non-performed panel would have identified the mutation for that patient.

Data could be available on reasonable demand.

## Results and Discussion

### Small Panels Are Better Than Hotspot Analysis

We first looked at the contribution of small panels (in-house and commercial panels) compared to hotspots.

**Figure 1:**
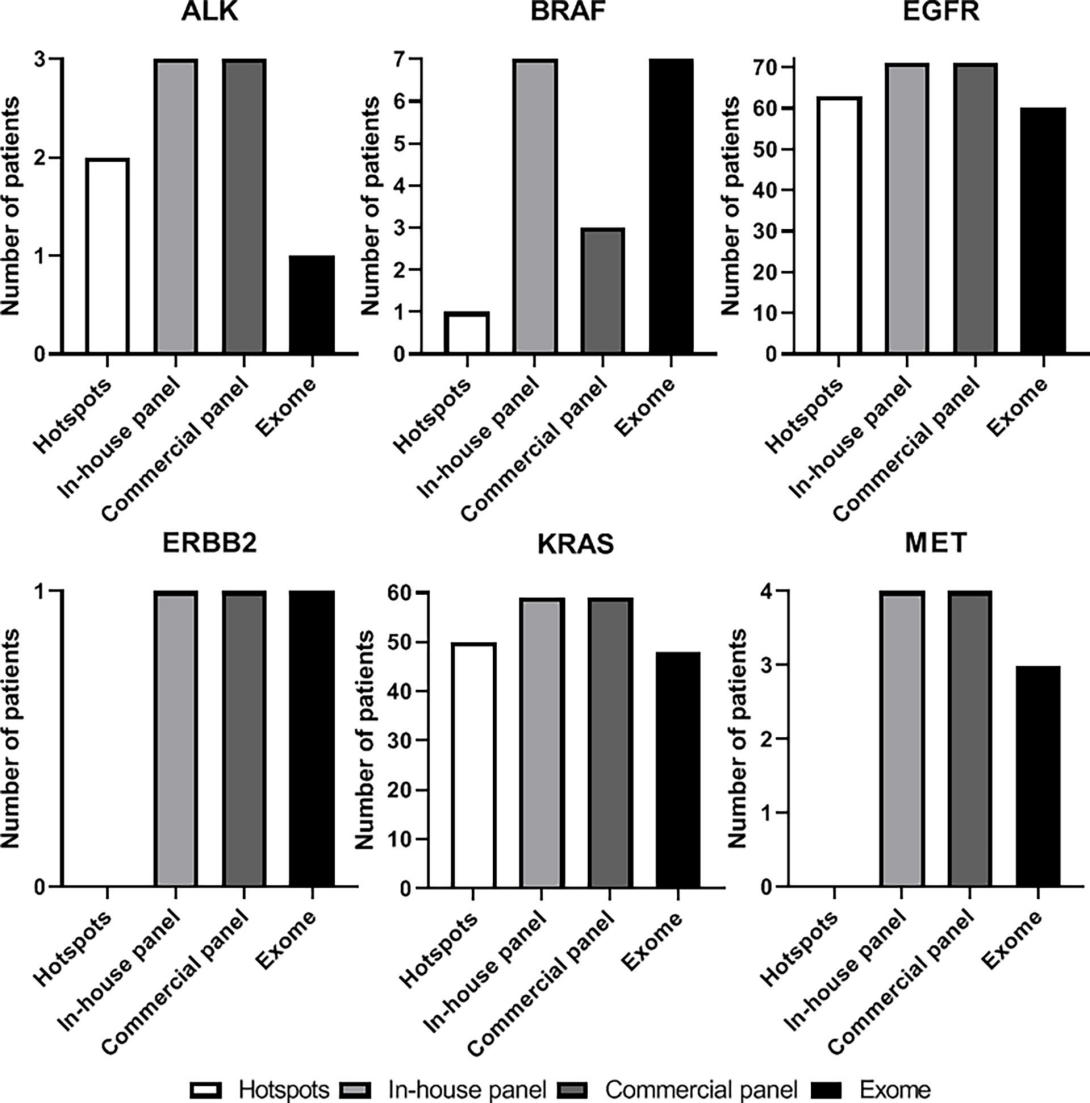
Number of patients with a mutation found on *ALK*, *BRAF*, *EGFR*, *ERBB2*, *KRAS* and *MET* genes according on the technic used: hotspot analysis (white), in-house panel (light grey), commercial panel (dark grey), or exome analysis (black).

For the six most frequently mutated genes (*ALK, BRAF, EGFR, ERBB2, KRAS* and *MET*), small panels found a greater number of mutations than hotspots. Mutations were found in 145 and 141 patients with small panels (CGFL and commercial panel respectively), and in 116 patients with the hotspots (**Figure 1**).

Regarding *EGFR*, small panels highlighted 9 mutations in 8 more patients than hotspots (**Supplementary Table 1**).

For the L858R mutation and the Exon20 insertions that were missed at the initial diagnosis, the molecular testing had been performed in an external laboratory. However, the molecular testing that missed the G719A mutation was performed in our laboratory, on the same sample as the one used for exome analysis.

In terms of therapeutic impact, it was important to know whether the mutations that were not found at initial diagnosis by hotspot analysis, were shown to be sensitive to EGFR tyrosine kinase inhibitors (TKIs). Afatinib and osimertinib have shown clinical benefit in patients with G719A and S768I (4, 5). The same applies for the L858R mutation, which is sensitive to most EGFR TKIs. Conversely, mutations in exon 20 (D770_N771 (insG), P772_H773 (insHA) and V769L) seem to be predictive markers of TKI resistance (6, 7). Nowadays, promising targeted therapies against Exon20ins, such as amivantamab (8), mobocertinib (9) and poziotinib (10), have shown strong responses in those patients. The impact of EGFR TKIs on R832C, A840T and G917R is currently unknown.

Regarding *KRAS*, 2 mutations were not found by hotspot analysis. One of these two mutations was a G12C, which has recently become targetable with sotorasib (11). The G12C mutation was not found initially, because the diagnosis was made on a liquid biopsy, which has lower sensitivity than solid biopsy. The other mutation was a G13C that was not covered by the hotspot analysis.

### What is the Benefit of Exome Analysis?

After these examinations, we sought to determine the benefit that could be yielded by exome analysis, as compared to small panels.

**Figure 2:**
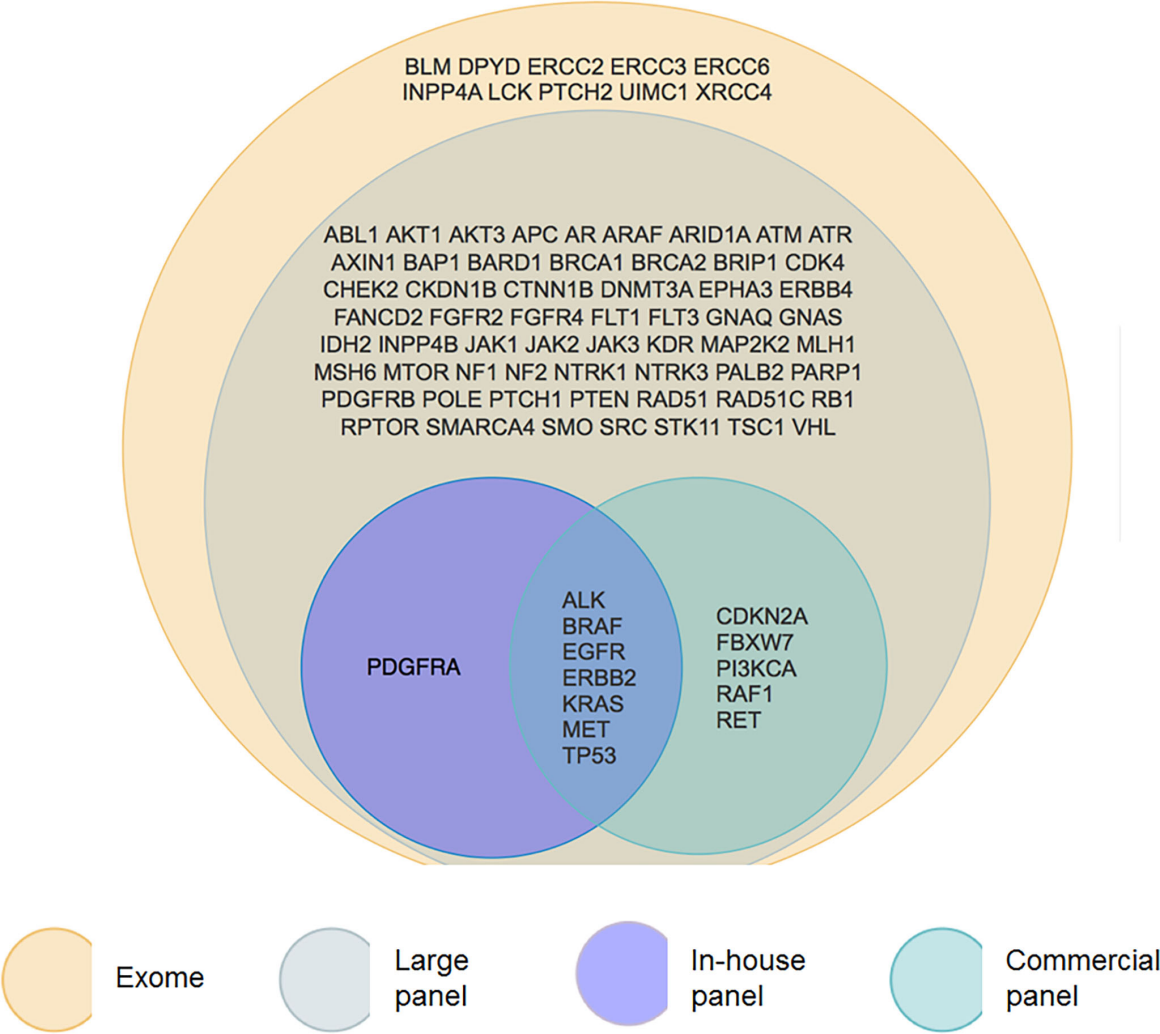
Venn diagram of presenting genes with a mutation found by each panel. We can observe that exome (yellow) contains large panel (grey) that contains itself smaller panels. In-house panel (purple) and commercial (green) panel have some common genes but have also some differences in gene content.

Overall, exome analysis found mutations in 82 genes (**Figure 2**). Among these, 72 are explored by the FoundationOne panel. The commercial panel and the in-house panel found mutations in 8 and 12 of these genes respectively. The most frequently mutated genes (*ALK, BRAF, EGFR, ERBB2, KRAS, MET*) are studied by all the panels.

**Figure 3:**
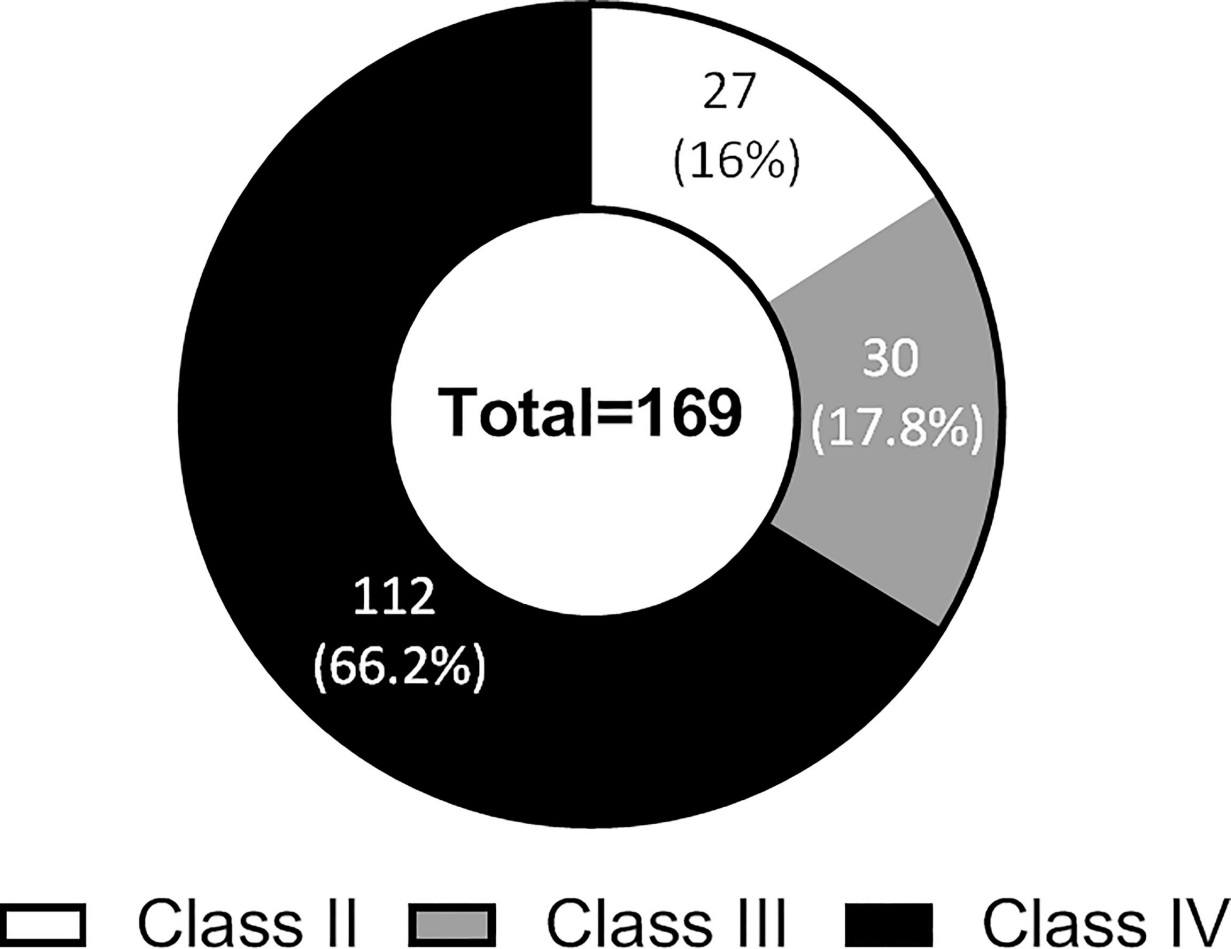
ESCAT class distribution of the mutations found by exome analysis only in genes not contained in small panels.

The exome analysis made it possible to highlight mutations on hundreds of genes, whose impact on patient management remains uncertain. Mutations exclusively found by the exome analysis were categorized according to the ESCAT classification (details of ESCAT classifications are reported in **Supplementary Table 2**). Sixteen percent (n = 27) were class II mutations, 17.8% (n = 30) class III mutations, and 66.2% (n = 112) were class IV mutations (**Figure 3**). There was no class I mutation found only by exome analysis, as these mutations are all explored by smaller panels.

It should be underlined that all *KRAS* mutations were considered as class III mutations since no targeted treatment had been approved at the time of those patients molecular screening.

### What Are the Limitations of Large Panel or Exome Analysis?

Exome analysis made it possible to highlight some mutations that are not explored by other panels. However, exome analysis has some limitations in terms of its sensitivity.

For most the common mutations, exome analysis has a slightly lower efficiency than small panels (**Figure 1**). It found fewer *ALK, EGFR, KRAS* and *MET* mutations. Conversely, for *BRAF*, it showed the same efficacy as the CGFL panel, and even showed superior efficacy compared to the commercial panel.

Large panels highlighted substantially more class II, III and IV mutations, which, for the most part, were not targetable at the time of first line treatment. However, large panels were slightly less efficient for class I mutations (**Supplementary Figure 1**).

Nevertheless, it is becoming crucial to be aware of the existence of some mutations, even at an early stage of the disease, because of the potential impact of these mutations on other therapies. As suggested by the IMMUNOTARGET trial (12), the clinical activity of immunotherapy is lower in the presence of actionable alterations. With immunotherapies being developed at earlier stages of the disease, even in neoadjuvant or adjuvant strategies, exhaustive molecular status of tumors will be needed as soon as possible after the diagnosis. The same applies to *EGFR* mutations, with osimertinib improving the disease-free survival in adjuvant situations (13).

Regarding *EGFR* mutations, 11 mutations found with small targeted panels were not found by the exome analysis (**Supplementary Table 3**). These were mainly exon 19 deletions (n = 9), but also a G735S and an Exon 20 insertion (D770_delinsDNPY). Exon 19 deletions are widely known to be sensitive to EGFR TKIs. In contrast, D770_delinsDNPY is rather predictive of resistance to EGFR TKIs. The impact of therapies targeting EGFR on G735S remains unknown as yet.

For *KRAS* mutations, 11 were not found by the exome analysis. Among these, 3 are potentially targetable G12Cs, whereas the details of the *KRAS* mutation were not known for 4 of them.

It should not be forgotten that large NGS and WES are very expensive techniques that also require a long time for analysis, and they cannot be obtained as rapidly as smaller targeted panels. Considering these results, large sequencing techniques could be reserved for implementation at disease progression, while small panels seem to be sufficient to guide the choice of first line treatment (**Supplementary Figure 2**).

## Conclusion

At the initial diagnosis of metastatic NSCLC, targeted gene panels should be preferred to guide the choice of first line treatment. They are more sensitive than larger panels for ESCAT level I mutations, and results can be obtained faster, and at a lower cost.

Large panels or WES can be useful at disease progression, to widen the treatment possibilities by highlighting potentially targetable mutations, and enabling inclusion of patients in clinical trials. Large panels or WES are able to detect uncommon mutations on frequently mutated genes, and can highlight mutations on genes that are not covered by targeted panels.

## Data Availability Statement

The original contributions presented in the study are included in the article/**Supplementary Material**. Further inquiries can be directed to the corresponding author.

## Ethics Statement

Patients were all included in the EXOMA (NCT02840604) or ALCAPONE (NCT02281214) trials. This study on patient samples was conducted in accordance with the Declaration of Helsinki and approved by the Ethics Committee of the Georges-François Leclerc Cancer Center (Dijon, France) under the number 00010311, and by the Consultative Committee of Burgundy (Dijon, France) for the Protection of Persons Participating in Biomedical Research (Comité Consultatif de Protection des Personnes en Recherche Biomédicale de Bourgogne). The patients/participants provided their written informed consent to participate in this study.

## Author Contributions

Conceptualization: RB. Methodology: RB, JN, and CK. Validation: RB, JN, and CK. Formal analysis: LD. Investigation: LD, JN, and CK. Data curation: LD, JN, and CK. Writing - original draft: LD and JN. Writing - review & editing: CK and RB. Visualization: LD and RB. Supervision: RB. Project administration: RB. All authors contributed to the article and approved the submitted version.

## Conflict of Interest

The authors declare that the research was conducted in the absence of any commercial or financial relationships that could be construed as a potential conflict of interest.

## Publisher’s Note

All claims expressed in this article are solely those of the authors and do not necessarily represent those of their affiliated organizations, or those of the publisher, the editors and the reviewers. Any product that may be evaluated in this article, or claim that may be made by its manufacturer, is not guaranteed or endorsed by the publisher.

## Supplementary Material

Supplementary Figure 1 ESCAT class distribution of the mutations found according to the panel used.

Supplementary Figure 2 Positioning of small panels and large NGS in the management of NSCLC.
