# Supplementary figures and images for: Is There a Role for Large Exome Sequencing in the Management of Metastatic Non-Small Cell Lung Cancer: A Brief Report of Real Life
Source: Front Oncol. 2022 Mar 7;12:863057. doi: 10.3389/fonc.2022.863057 (PMC8940536; doi:10.3389/fonc.2022.863057)

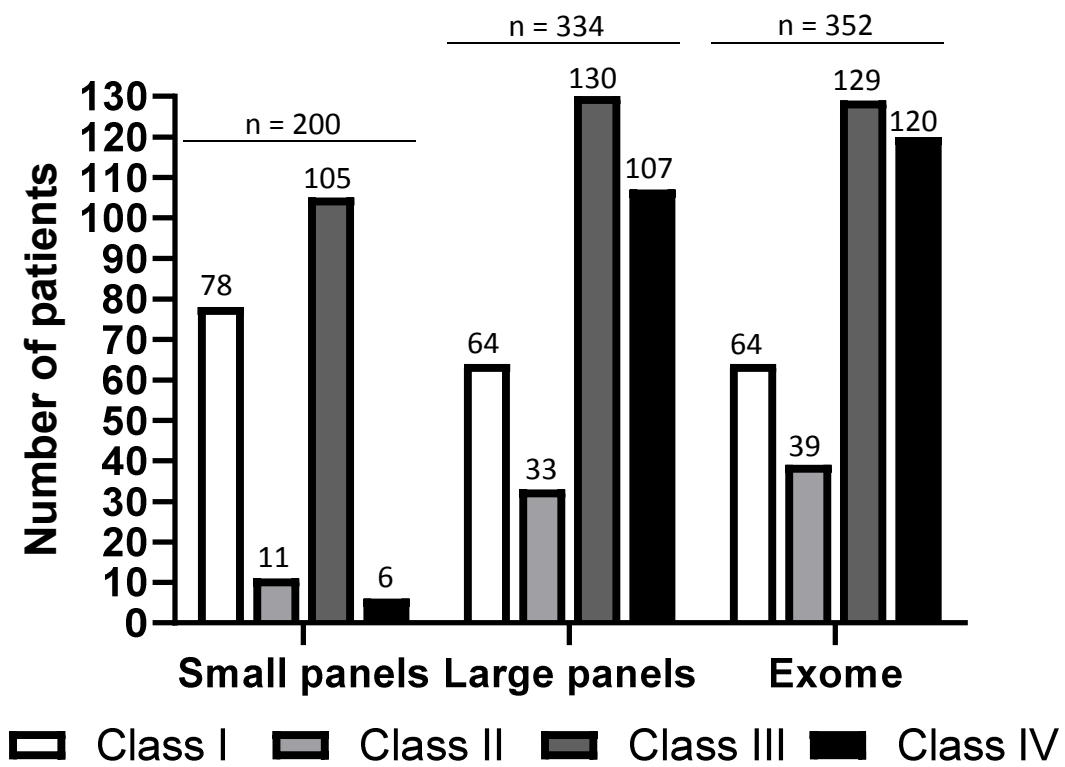

Supplementary Figure 1

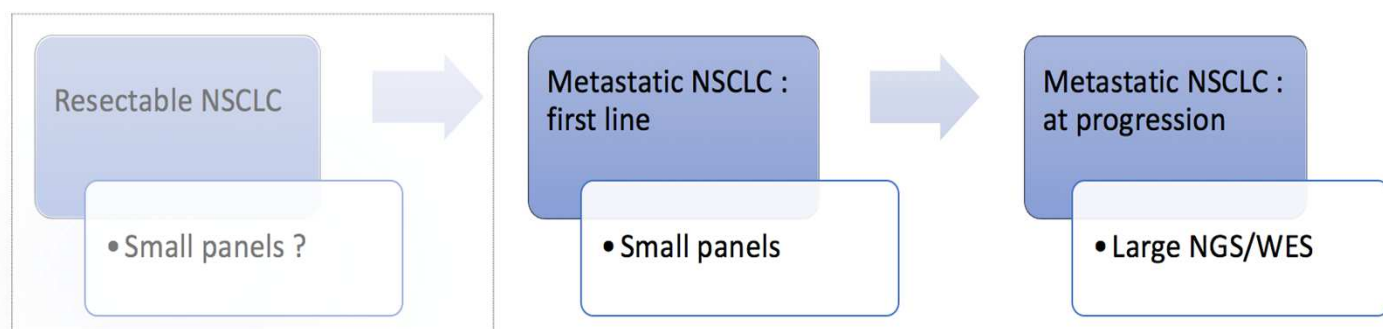

Supplementary Figure 2

Supplement: Supplementary Figure 1 — ESCAT class distribution of the mutations found according to the panel used. [file DataSheet_1.pdf]
