# Supplementary material for: Is There a Role for Large Exome Sequencing in the Management of Metastatic Non-Small Cell Lung Cancer: A Brief Report of Real Life
Source: Front Oncol. 2022 Mar 7;12:863057. doi: 10.3389/fonc.2022.863057 (PMC8940536; doi:10.3389/fonc.2022.863057)
Supplement: Supplementary file 2 [file Table_1.docx]

Supplementary Table 1: EGFR mutations highlighted by small panels and by exome analysis, but not found by hotspot analysis

| Exon | Nucleotide variation | Amino acid variation | Impact | Presumed clinical impact |
| --- | --- | --- | --- | --- |
| 18 | c.2156G>C | p.(Gly719Ala) | Activating | Sensitivity to Afatinib and Osimertinib |
| 20 | c.2303G>T | p.(Ser768I) | Activating | Sensitivity to Afatinib and Osimertinib |
| 20 | c.2305G>T | p.(Val769Leu) | Activating | Resistance to EGFR TKI |
| 20 | c.2310_2311insGGG | p.(Asp770_Asn771insGly) | Activating | Resistance to EGFR TKI |
| 20 | c.2314_2315insCCCACG | p.(Pro772_His773insHisAla) | Activating | Resistance to EGFR TKI |
| 21 | c.2494C>T | p.(Arg832Cys) | Activating | Unknown |
| 21 | c.2518G>A | p.(Ala840Thr) | Unknown | Unknown |
| 21 | c.2573T>G | p.(Leu858Arg) | Activating | Sensitivity to EGFR TKI |
| 23 | c.2749G>A | p.(Gly917Arg) | Unknown | Unknown |

TKI: Tyrosine Kinase Inhibitors
