# Supplementary material for: Is There a Role for Large Exome Sequencing in the Management of Metastatic Non-Small Cell Lung Cancer: A Brief Report of Real Life
Source: Front Oncol. 2022 Mar 7;12:863057. doi: 10.3389/fonc.2022.863057 (PMC8940536; doi:10.3389/fonc.2022.863057)
Supplement: Supplementary file 3 [file Table_2.docx]

Supplementary Table 2: ESMO Scale For Clinical Actionability of molecular Targets

| ESCAT level I | Drug match is associated with improved outcome in clinical trials |
| --- | --- |
| ESCAT level II | Drug match is associated with antitumor activity, but magnitude of benefit is unknown |
| ESCAT level III | Drug match suspected to improve outcome based on clinical trial data in other tumor type(s) or with similar molecular alteration |
| ESCAT level IV | Preclinical evidence of actionability |
