# Supplementary material for: Is There a Role for Large Exome Sequencing in the Management of Metastatic Non-Small Cell Lung Cancer: A Brief Report of Real Life
Source: Front Oncol. 2022 Mar 7;12:863057. doi: 10.3389/fonc.2022.863057 (PMC8940536; doi:10.3389/fonc.2022.863057)
Supplement: Supplementary file 4 [file Table_3.docx]

Supplementary Table 3: EGFR mutations not found by exome

| Exon | Nucleotide variation | Amino acid variation | Number of mutations | Impact | Presumed clinical impact |
| --- | --- | --- | --- | --- | --- |
| 19 | In-frame deletion | | 9 | Activating | Sensitivity to EGFR TKI |
| 19 | c.2203G>A | p.(Gly735Ser) | 1 | Activating | Unknown |
| 20 | c.2310delinsGACAATCCCAAG | p.(Asp770delinsAspAsnProLys) | 1 | Activating | Resistance to EGFR TKI |

TKI: Tyrosine Kinase Inhibitors
